# ImmunExplorer (IMEX): a software framework for diversity and clonality analyses of immunoglobulins and T cell receptors on the basis of IMGT/HighV-QUEST preprocessed NGS data

**DOI:** 10.1186/s12859-015-0687-9

**Published:** 2015-08-12

**Authors:** Susanne Schaller, Johannes Weinberger, Raul Jimenez-Heredia, Martin Danzer, Rainer Oberbauer, Christian Gabriel, Stephan M. Winkler

**Affiliations:** 1 University of Applied Sciences Upper Austria, Hagenberg Campus, Bioinformatics Research Group, Softwarepark 13, Hagenberg, 4232 Austria; 2 Red Cross Transfusion Service of Upper Austria, Krankenhausstrasse 7, Linz, 4020 Austria; 3 Ludwig Boltzmann Institute for Experimental and Clinical Traumatology, Donaueschingenstrasse 13, Vienna, 1200 Austria; 4 Elisabethinen Hospital, Fadingerstrasse 1, Linz, 4020 Austria; 5 Medical University of Vienna, Department of Nephrology, Spitalgasse 23, Vienna, 1090 Austria

## Abstract

**Background:**

Today’s modern research of B and T cell antigen receptors (the immunoglobulins (IG) or antibodies and T cell receptors (TR)) forms the basis for detailed analyses of the human adaptive immune system. For instance, insights in the state of the adaptive immune system provide information that is essentially important in monitoring transplantation processes and the regulation of immune suppressiva. In this context, algorithms and tools are necessary for analyzing the IG and TR diversity on nucleotide as well as on amino acid sequence level, identifying highly proliferated clonotypes, determining the diversity of the cell repertoire found in a sample, comparing different states of the human immune system, and visualizing all relevant information.

**Results:**

We here present IMEX, a software framework for the detailed characterization and visualization of the state of human IG and TR repertoires. IMEX offers a broad range of algorithms for statistical analysis of IG and TR data, CDR and V-(D)-J analysis, diversity analysis by calculating the distribution of IG and TR, calculating primer efficiency, and comparing multiple data sets. We use a mathematical model that is able to describe the number of unique clonotypes in a sample taking into account the true number of unique sequences and read errors; we heuristically optimize the parameters of this model. IMEX uses IMGT/HighV-QUEST analysis outputs and includes methods for splitting and merging to enable the submission to this portal and to combine the outputs results, respectively. All calculation results can be visualized and exported.

**Conclusion:**

IMEX is an user-friendly and flexible framework for performing clonality experiments based on CDR and V-(D)-J rearranged regions, diversity analysis, primer efficiency, and various different visualization experiments. Using IMEX, various immunological reactions and alterations can be investigated in detail. IMEX is freely available for Windows and Unix platforms at http://bioinformatics.fh-hagenberg.at/immunexplorer/.

**Electronic supplementary material:**

The online version of this article (doi:10.1186/s12859-015-0687-9) contains supplementary material, which is available to authorized users.

## Background

Immune repertoire is a term that is commonly used in immunology to describe the level of diversity and clonality of B and T cell antigen receptors, the immunoglobulins (IG) or antibodies and T cell receptors (TR). These cells encode an humongous variety of receptors that are capable of recognizing any organic macromolecule of biological relevance. The main process for the generation of the antigen receptors is called receptor rearrangement and is very similar for B and T cells: Every antigen receptor consists of two different chains that are responsible for antigen recognition, namely the *α* (TRA) and *β* (TRB) chain, and *γ* (TRG) and *δ* (TRD) for *α* *β* and *γ* *δ* TR, the immunoglobulin heavy chain (IGH), and one of two different immunoglobulin light chains (IGK, IGL) for the immunoglobulins or antibodies. IGH and TRB V domains are encoded by three different gene segments: variable (V), diversity (D) and joining (j); IGK, IGL and TRA V domains are encoded by two gene types, V and J [1]. A human genome in germline confirmation comprises alleles for every gene [2]. During B and T cell development the cells rearrange the genes so that there is only one V gene and one J gene per rearrangement (and usually one D for IGH and TRB, but several for TRD), and J element per functional exon. An important principle called allelic exclusion ensures that only one receptor specificity is expressed per B or T cell.

The human adaptive immune system has a strong impact on human health. Its efficiency is fundamentally reliant upon antigen receptor diversity; a restricted repertoire is in many cases unable to recognize the full variety of pathogens. In addition, an immune response as well as certain diseases lead to clonal expansions of B and T cells depending on their receptor specificity. Therefore, analyzing and understanding the repertoire is highly beneficial for research issues as well as to optimize medical treatment of patients [3].

Today’s most advanced techniques in immune repertoire analysis are based on next-generation sequencing (NGS) [4] that produces huge amounts of data. Currently, there exist various analysis and visualization tools for system immunology with different focuses such as, for example, MiTCR [5], Decombinator [6], IMGT/HighV-QUEST [7], IgBLAST [8], ImmunTraCkeR [9], immunoSEQ [10], IgAT Tool [11], and IgTree [12].

Some of those tools are focused on calculating a wide range of statistics (e.g., IgAT), performing alignments to facilitate analysis of the immunglobulin variable domain sequences (e.g., IgBLAST) or generating lineage trees from immunoglobulin variable region gene sequences (e.g., IgTree). All those tools are based on analyzing the B cell repertoire, while others enable detailed research on the T cell repertoire: For example, ImmunTraCkeR determines V-J rearrangements and sets the main focus on the cell immune repertoire diversity. MiTCR offers a fast CDR3 algorithm and a PCR two-stage approach for correcting sequencing errors. ImmunoSEQ mainly places emphasis on statistical analysis and visualization of IG and TR data.

Whereas most of these tools/frameworks are focused on one cell type or on one specific type of analysis, our here presented framework **IMEX** has been designed for comprehensive, in-depth analysis of human antigen IG and TR repertoires based on NGS data. IMEX contains algorithms for gaining more knowledge about the diversity on different sequence levels based on IMGT/HighV-QUEST analysis outputs [7, 13]. In the context of the calculation of clonality, IMEX users are able to define how to calculate sequence clonality and to compare diversity and clonality of various samples. A primer efficiency analysis enables the investigation of primer matching frequencies in PCR experiments. IMEX also includes V-(D)-J gene combination algorithms and additionally offers a wide range of visualization methods for gaining essential insights in the human adaptive immune system.

## Implementation

IMEX includes algorithms and statistical analyses for determining descriptive statistics about sequence functionality and V-(D)-J rearranged region frequency, calculating clonality of cells, estimating diversity of the cell spectrum, and visual representation of various gene/allele combinations. IMEX has been designed for analyzing and summarizing NGS-based IG and TR data derived from IMGT®;. IMGT/HighV-QUEST is a NGS high-throughput analysis portal for IG and TR, and so far the only one available online [7, 13]. IMGT/HighV-QUEST uses the same algorithms as IMGT/V-QUEST [14] with integrated IMGT/JunctionAnalysis [15], provides 11 compressed output files that contain information about variable (V), diverse (D), and joining (J) gene arrangements (V-(D)-J), identification and characterization of new alleles, detailed analysis of the junction (IMGT/JunctionAnalysis results), and additional information of mutations. IMEX uses these processed files as input for statistical analyses. Sample comparisons, clonotype tracking, and variety analysis are also included in IMEX. IMEX is written in C# and is freely available at http://bioinformatics.fh-hagenberg.at/immunexplorer/. In the following paragraphs we give detailed descriptions of the analysis methods implemented in IMEX.

### Preprocessing methods for the IMGT/HighV-QUEST submission

The IMGT/HighV-QUEST online portal enables uploading and processing of up to 500,000 sequences, therefore preprocessing methods have been developed in IMEX: FASTA files can be split into several files (using a user-defined threshold for the size of these files) to prepare the upload to the IMGT®; information system; after uploading to IMGT/HighV-QUEST [16] at IMGT®;, the international ImMunoGeneTics information system®; (http://www.imgt.org) [17] and analyzing, the compressed output files can be merged to one compressed data file. This file includes all information that is needed for determining overall statistics of the IG and TR clonotypes, frequencies, diversity and V-(D)-J rearranged region frequencies using IMEX.

### Descriptive statistic analyses

**Fig. 1:**
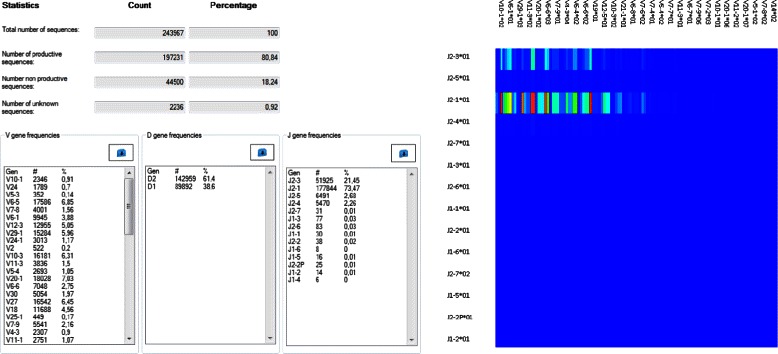
Sequence basics analysis in IMEX. The total number and relative frequencies of productive, unproductive, and unknown sequences are shown. V, D and J gene lists can be displayed and downloaded for further analyses

IMEX enables a wide range of statistical analyses of IG and TR data. Lists of V, D, and J gene occurrences containing the total amounts and relative frequencies of these genes are calculated as well as the total amounts of the productive, unproductive, and unknown sequences (see Fig. 1). Sequences, for which no alignment result was found, are reported, but not considered later when it comes to further calculations in IMEX. Additionally, pie charts can be generated to gain more insights about the productive and unproductive B and T cell arrangements of the human adaptive immune system. All statistical calculations can be downloaded as text files and used for further calculations.

### Clonality analysis

The clonality of the IG and TR based on theV-(D)-J rearranged regions, the CDR3 sequences, and/or the nucleotide sequence of the whole amplicon provides additional information. Clonal expansion is related to the level of somatic proliferation of single B or T cell clonotypes triggered by various immunological reactions. In IMEX, the calculation of clonality can be defined by the user by choosing the amino acid or the nucleotide sequence or the V-(D)-J rearranged regions. IMEX enables the calculation of the clonality based on the three complementarity determining regions (CDR), namely CDR1, CDR2, and CDR3. CDR3, the most variable CDR, can be found in the junction of the rearranged V-(D)-J regions. The number of clonotypes can also be determined using the nucleotide sequence of the whole read of the V-(D)-J rearranged region. Total numbers and relative frequencies of the clonotypes are given in tabular view; these lists can be exported and used for further analyses.

### Diversity analysis

The diversity of an antigen receptor repertoire is calculated by analyzing the unique clonotypes of IG and TR in all sequences.

In the literature, several different ways to define the term diversity can be found [18]; IgAT, for example, calculates the clonotypic diversity as clonotypes per productive sequences and the sequence diversity as unique sequences per productive sequences [11]. IMEX calculates sequence diversity using a more elaborated data mining approach [19] based on the most variable region, the CDR3 [7]:

To empirically calculate the diversity in IG or TR data, we randomly choose *n* out of *N* CDR3 sequences (*r* *a* *n* *d*(*n*,*N*)) in the sample and determine the number of unique clonotypes (*c*_*unique*_) in these *n* sequences. This *c*_*unique*_(*n*) is calculated for increasing numbers of *n*, for example for *n*={0,1000,2000,3000,…}, and so we get the calculated diversity *d* *i* *v*_*calc*_(*n*) in *n* sequences:

(1) $$\begin{array}{@{}rcl@{}}  div_{calc}(n)= c_{unique}(rand(n,N)) \end{array} $$

**Fig. 2:**
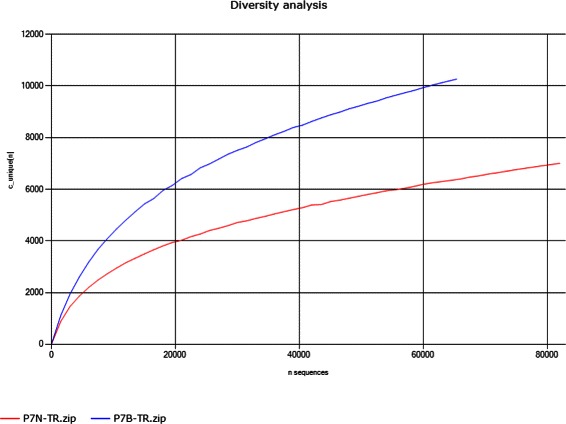
Diversity analysis in IMEX. The diversity (*d* *i* *v*_*calc*_(*n*)) of two different samples of one patient (P7) is shown. We see that sample P7N is more diverse than sample P7B. There is an increase of the number of unique clonotypes in the beginning but the more sequences we use for calculating the number of unique clonotypes the more the curve tends to become linear

This calculation is repeated five times for each *n* and the number of unique clonotypes *c*_*unique*_ is averaged. Examples are shown in Fig. 2.

We assume that there is a certain amount of unique clonotypes in the sample, and the more amino acid sequences we draw from the sample, the more the number of unique sequences will converge to the true number of unique clonotypes. Additionally, we have to keep in mind that the more sequences we draw, the more unique sequences we will see due to read errors. This is why we assume that the number of unique sequences (seen in *n* randomly drawn sequences) can be modeled as

(2) $$\begin{array}{@{}rcl@{}}  div_{mod}(n)= a * (1-e^{-b*n})+k*n \end{array} $$

where *a* is the true number of unique clonotypes and *k* is the fraction of unique sequences caused by read errors.

**Fig. 3:**
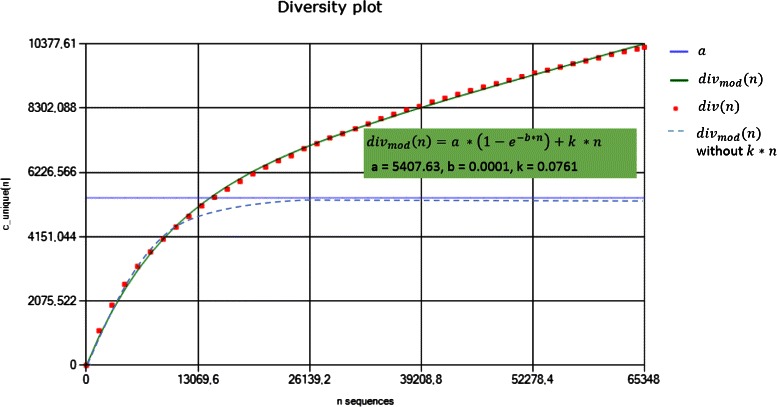
Evaluation of the diversity model with parameter optimization. The parameters of the diversity model (green curve) are optimized using evolution strategies. The red spots are calculated as explained in Eq. 1. The dashed light blue curve describes the number of unique clonotypes without sequencing errors. The value *a* corresponds to the true number of unique clonotypes in the sample

The parameters *a*, *b*, and *k* of the here proposed model are optimized so that they fit the empirically calculated diversity *d* *i* *v*_*calc*_ using evolution strategies [20]. The so optimized *a* in the model corresponds to the total number of unique clonotypes in the multiplex PCR as shown in Fig. 3.

### V-(D)-J visualizer

IMEX provides an algorithm for visualizing various V-(D)-J rearranged region combinations. All V-J, V-D, J-D and V-(D)-J gene and/or allele combinations are determined in the data sample. The framework contains several different graphical representation possibilities to visualize the total gene and allele frequencies; frequency histograms, heat maps, and bubble charts can be created and enable detailed visualizations of the state of the investigated receptor repertoire. Gene and allele frequencies can be sorted by gene names so that results for different samples can be compared easily. A frequency threshold can be used to filter specific genes and alleles.

IMEX also offers the download of all B and T cell genes and alleles listed in the IMGT information system®; for the species *Homo Sapiens*. For the visualization of the V-(D)-J rearranged region distributions we have first calculated a list of all possible V-(D)-J combinations; all V-(D)-J combinations of a sample are determined and mapped on the full spectrum of all known V-(D)-J rearranged regions. This enables an accurate approach to compare various samples on gene or allele level.

### PCR primer matching

IMEX includes a feature for analyzing primer efficiency. Primer sets used for multiplex rearranged V-(D)-J regions PCR amplification can be imported (see Additional file 1: Primer lists for TRB and IGH). This primer matching algorithm searches for the exact sequences in the IMGT aligned sequences and returns the relative frequency of each primer in the imported primer sets. This enables the optimization of the efficiency in multiplex PCR.

### Comparison analysis

The comparison of various two or more samples with respect to the clonality of the IG and TR repertoire is an essential analysis feature in IMEX:

- Pairwise CDR3 Clone Comparer: IMEX is capable of generating a list of unique CDR3 clonotypes of each data sample and searching the top *c*_*unique*_ clonotypes from one sample in the other sample. Each clonotype is assigned a randomly chosen color and matched clonotypes are shown in the same color.
- Multiple CDR3 Clone Comparer: The multiple comparison algorithm generates the top *c*_*unique*_ clonotypes in each given data sample and searches for all so collected clonotypes in data samples. IMEX also contains a visualization and tabular view to compare overlapping multiple data samples according to CDR3.
- Multiple V-(D)-J Clone Comparer: As clonality can not only be defined over the CDRs but also over the V-(D)-J rearranged regions, IMEX also offers a multiple V-(D)-J Clone Comparer. The functionality is implemented in analogy to the Multiple CDR3 Clone Comparer.

### Approval of ethics committee and consent

Informed written consent was obtained from all participating individuals according to the Declaration of Helsinki. Ethical approval for the sample collection used here was obtained from the Ethical Committee of Upper Austria (no. E-9-12, Jan 21^*st*^, 2013).

## Results and discussion

Here we demonstrate the analysis of NGS data of a proband whose immune spectrum showed highly abundant clonal Expansion over a longer time period. Using analysis methods provided by IMEX we found two cytotoxic T cell clonotypes (CD8+) that are highly abundant and can be constantly observed over several months. The data sets have been obtained using PCR (Biomed 2 primer panels for gDNA amplification) of the IGH and TRB loci [21] followed by next-generation sequencing (Illumina Miseq sequencer).

**Fig. 4:**
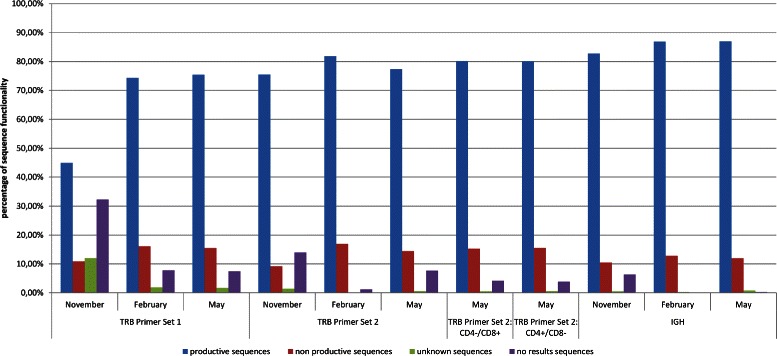
Sequence analysis for proband p78690 using IMEX. This figure shows the relative frequencies of the productive, unproductive, unknown, and unidentified sequences. We see that the number of sequences that cannot be aligned to the target locus is low, which indicates a high number of unspecific amplicons

We took blood samples of the proband p78690 at three different time points (November 2013 (T1), February 2014 (T2), and May 2014 (T3)); for every time point we generated three data sets, one of the IGH chain and two of the TRBV chain (primer sets 1 and 2). After having analyzed the data using IMGT/HighV-QUEST online (http://www.imgt.org), we performed statistical sequence analysis of the so generated data sets, the results are given in Tables 1 and 2. These data form the basis of a first, general overview of the IG and TR repertoires, shown in Fig. 4.

**Table 1:** Basic analysis in IMEX of the IMGT/HighV-QUEST sequence alignments for the TR using primer set 1 and 2 of proband p78690

Proband: p78690	TRB primer set 1			TRB primer set 2			TRB primer set 2:	TRB primer set 2:
							CD4-/CD8+	CD4+/CD8-
	*T1*	*T2*	*T3*	*T1*	*T2*	*T3*	*T3*	*T3*
Productive	16,707	104,346	91,840	77,380	77,997	148,676	224,397	170,696
Unproductive	4018	22,534	18,739	9382	16,066	27,577	42,477	32,884
Unknown	4461	2639	1994	1424	67	1102	1403	1167
No result	11,989	10,879	9068	14,268	1162	14,694	11,498	8135
Total number of sequences	37,175	140,398	121,641	102,454	95,292	192,049	279,775	212,882

For each TR primer set (T cell receptor *β* chain amplification based on Biomed-2 primer sets) we have determined the total number of productive, unproductive, unknown and no result sequences. For primer set 2 at time point 3 we prepared an additional basic analysis for CD4-/CD8+ and CD4+/CD8- sorted cells

**Table 2:** Basic analysis of the IMGT/HighV-QUEST sequence alignment for the IGH. The analysis was done accordingly as described in Table 1

Proband: p78690	IGH		
	*T1*	*T2*	*T3*
Productive	196,479	105,767	201,582
Unproductive	24,770	15,513	27,748
Unknown	1105	314	1769
No result	14,915	156	620
Total number of sequences	237,269	121,750	231,719

**Fig. 5:**
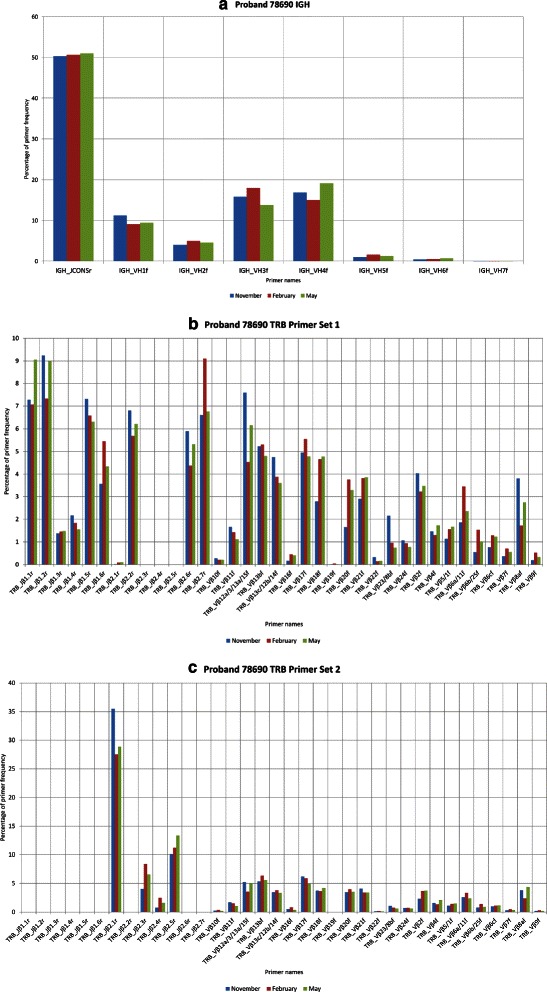
PCR primer analysis in IMEX. Figure **a** shows that no amplicons derived from primer IGHV7 and almost no amplicons from primer IGHV6 are found in the sample. Figures **b** and **c** show that these two amplifications differ in their primer compositions. While forward primers have the same frequencies at all time points, the frequencies of reverse primers vary significantly

We additionally tested the contribution of the multiplex primers to the total number of generated sequences by using the IMEX PCR primer matching algorithm for quality control of the PCR; the results of this analysis are shown in Fig. 5. There we see that no amplicons derived from primer IGHV7 subgroup and almost no amplicons from primer IGHV6 subgroup are found in the sample.

**Fig. 6:**
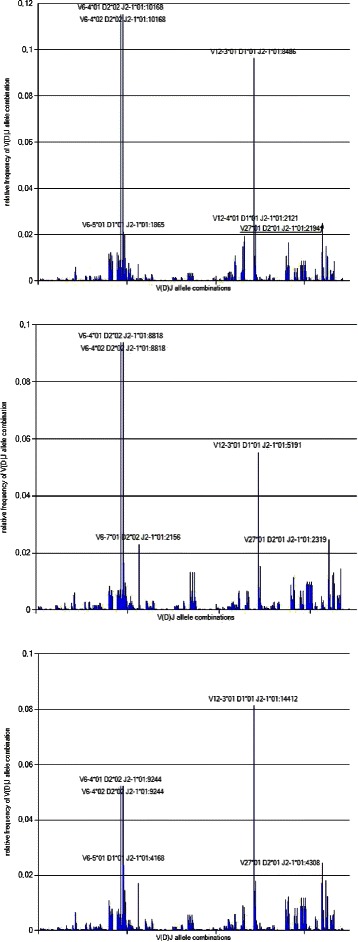
V-(D)-J visualizer in IMEX. Figures **a**-**c** represent the V-(D)-J gene spectrum of the proband for all three time points. Overall, the detected V-(D)-J gene combinations for all three time points look similar. Nevertheless, two highly abundant clonotypes can be observed (genes *V6–4 D2 J2* and *V12-3 D1 J2*), which need to be further investigated

**Fig. 7:**
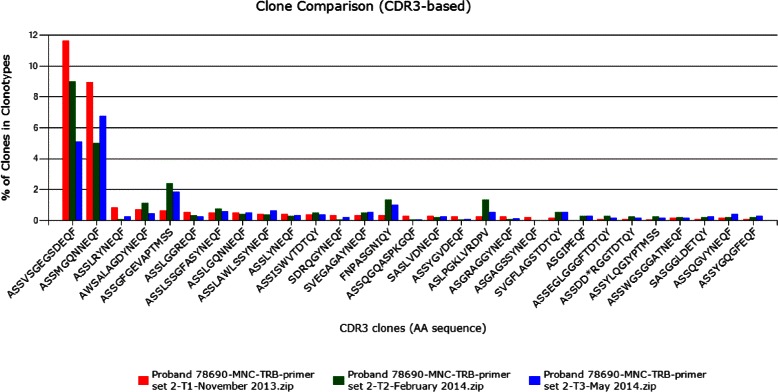
Tracking clonotypes over several time points. We here track clonotypes based on their CDR3 sequences over three different time points. In this particular example two clonotypes are highly expanded in all three time points (CDR3s: ASSVSGEGSDEQF, ASSMGQNNEQF)

In order to determine the variability of the IGH and TRB repertoire we analyzed and compared the V-(D)-J combinations of three different time points. As shown in Fig. 6, the TRB V-(D)-J rearrangement profile does not change over time, which means that the proband had no serious gene arrangement changes. We also see that there are two highly expanded V-(D)-J clonotypes that have to be analyzed in detail on gene level. Surprisingly we also found two highly abundant TRB CDR3s (AA) (ASSVSGEGSDEQF and ASSMGQNNEQF) for all three time points (see Fig. 7).

When unexpected expansions of clonotypes are detected, the next step is to investigate their cell types; more specifically, further insight can be gained by comparing CDR3 (AA) of CD8+ (cytotoxic) T cells (CD4-/ CD8+) and those of CD4+ (helper) T cells (CD4+/CD8-). We therefore separated CD8+ T cells from CD4+ T cells of the proband p78690 by fluorescence-activated cell sorting (FACS) based on the surface proteins CD4 and CD8 and compared the CDR3 (AA) at time point May to the following two T cell subsets. The results of this analysis are summarized in Table 3 where we see that the high frequency of CD8+ T cells is responsible for the high abundance of the two aforementioned clonotypes. An expansion of cytotoxic T cells is a common indication for intracellular viral or bacterial infections.

**Table 3:** Clonality comparison of the most abundant clonotypes based on CDR3 amino acid sequences in IMEX

CDR3 sequences	TRB Primer Set 2: CD4-/CD8+ T3	TRB Primer Set 2: CD4+/CD8- T3	MNC TRB Primer Set 2 T3
ASSMGQNNEQF	14.29447899	0.049037396	6.779536212
ASSVSGEGSDEQF	12.25748767	0.113766759	5.087625888
ASSGFGEVAPTMSS	5.681129492	0.002942244	1.856173462
FNPASGNIQY	2.506490689	0.003922992	1.020130731
ASLPGKLVRDPV	1.426455074	0.00245187	0.522809919
ASSLSSGFASYNEQF	1.366224477	0.00245187	0.570389587
ASSLGQNNEQF	1.048236852	0.002942244	0.481460922
AWSALAGDYNEQF	0.724263578	0.005394114	0.457671089
ASSLYNEQF	0.525988942	0.028932064	0.32286203
ASSLRYNEQF	0.414880324	0.002942244	0.233366941
SVGFLAGSTDTQY	0.062101113	0.860115924	0.549998301
SASGGLDETQY	0.003366928	0.398674029	0.240164037
ASSDD*RGGTDTQY	0.078187545	0.24273511	0.150102523
SVEVILDAGEQF	0.028431835	0.201543697	0.138207606

We compared the top 20 clonotypes of the unsorted (mononuclear) and the sorted (CD4-/CD8+ and CD4+/CD8-) sample of the TRB primer set 2 timepoint 3. By using this method we could identify that the most expanded T cells in the proband p78690 belong to the T cell subtype CD4-/CD8+ (surface marker for cytotoxic T cells)

## Conclusion

IMEX, a user-friendly tool for analyzing and visualizing IG and TR repertoires based on NGS data, has been presented in this paper. IMEX offers several algorithms for analyzing the clonality and diversity on multiple levels such as V-(D)-J arrangement, CDR, and nucleotide sequences of the whole reads. Moreover, it also provides features for analyzing primer efficiency. IMEX includes various visualization possibilities such as pie charts, histograms, line charts, bubble charts, and heat maps. We have shown that IMEX can be used for visualizing and comparing various aspects of the state of human adaptive immune repertoires.

The software framework IMEX was initially planned for analyzing and further processing IMGT/HighV-QUEST output files for gDNA-based sample preparation. During the development and implementation of IMEX, the community forged ahead in the field of immune repertoire sequencing, therefore we are currently extending the functionalities of IMEX. Algorithms and features for new cDNA sample preparation technologies i.e., single molecule barcoding which is able to reduce PCR bias will be implemented and extended in thenear future.

In addition, we plan to extend our analyses to other IG (IGK, IGL) and TR loci (TRA, TRG and TRD). Medium-term we are aiming to integrate a machine learning approach (based on algorithms implemented in in HeuristicLab (http://dev.heuristiclab.com/) [22]) that can classify immune status of patients with distinct diseases (e.g., bone marrow stem cell transplantation and minimal residual disease).

IMEX is freely available as GUI for Windows platforms and also as command line version for Windows/Linux and Unix systems and can be downloaded at http://bioinformatics.fh-hagenberg.at/immunexplorer/.

## Availability and requirements

- **Project Name:** ImmunExplorer (IMEX)
- **Project Web-page:** http://bioinformatics.fh-hagenberg.at/immunexplorer/
- **Operating System:** Windows, Linux and Unix
- **Programming Language:** C#
- **Other requirements:** Microsoft.NET framework 4.0
- **License:** see License Agreement on IMEX website http://bioinformatics.fh-hagenberg.at/immunexplorer/

## Additional file

Additional file 1
**Primer lists for TRB and IGH.** (PDF 31.3 kb)
