# Supplementary material for: ImmunExplorer (IMEX): a software framework for diversity and clonality analyses of immunoglobulins and T cell receptors on the basis of IMGT/HighV-QUEST preprocessed NGS data
Source: BMC Bioinformatics. 2015 Aug 12;16:252. doi: 10.1186/s12859-015-0687-9 (PMC4531494; doi:10.1186/s12859-015-0687-9)
Supplement: Additional file 1 — Primer lists for TRB and IGH. (PDF 31.3 kb) [file 12859_2015_687_MOESM1_ESM.pdf]

Supplementary Material

Primer set 1 for T cell receptors (TR)

Primer Name	Receptor Type	Sequence
TRB_Vβ2f	TCRB	AACTATGTTTTGGTATCGTCA
TRB_Vβ4f	TCRB	CACGATGTTCTGGTACCGTCAGCA
TRB_Vβ5/1f	TCRB	CAGTGTGTCCTGGTACCAACAG
TRB_Vβ6a/11f	TCRB	AACCCTTTATTGGTACCGACA
TRB_Vβ6b/25f	TCRB	ATCCCTTTTTTGGTACCAACAG
TRB_Vβ6cf	TCRB	AACCCTTTATTGGTATCAACAG
TRB_Vβ7f	TCRB	CGCTATGTATTGGTACAAGCA
TRB_Vβ8af	TCRB	CTCCCGTTTTCTGGTACAGACAGAC
TRB_Vβ9f	TCRB	CGCTATGTATTGGTATAAACAG
TRB_Vβ10f	TCRB	TTATGTTTACTGGTATCGTAAGAAGC
TRB_Vβ11f	TCRB	CAAAATGTACTGGTATCAACAA
TRB_Vβ12a/3/13a/15f	TCRB	ATACATGTACTGGTATCGACAAGAC
TRB_Vβ13bf	TCRB	GGCCATGTACTGGTATAGACAAG
TRB_Vβ13c/12b/14f	TCRB	GTATATGTCCTGGTATCGACAAGA
TRB_Vβ16f	TCRB	TAACCTTTATTGGTATCGACGTGT
TRB_Vβ17f	TCRB	GGCCATGTACTGGTACCGACA
TRB_Vβ18f	TCRB	TCATGTTTACTGGTATCGGCAG
TRB_Vβ19f	TCRB	TTATGTTTATTGGTATCAACAGAATCA
TRB_Vβ20f	TCRB	CAACCTATACTGGTACCGACA
TRB_Vβ21f	TCRB	TACCCTTTACTGGTACCGGCAG
TRB_Vβ22f	TCRB	ATACTTCTATTGGTACAGACAAATCT
TRB_Vβ23/8bf	TCRB	CACGGTCTACTGGTACCAGCA
TRB_Vβ24f	TCRB	CGTCATGTACTGGTACCAGCA
TRB_Jβ1.1r	TCRB	CTTACCTACAACTGTGAATCTGGTG
TRB_Jβ1.2r	TCRB	CTTACCTACAACGGTTAACCTGGTC
TRB_Jβ1.3r	TCRB	CTTACCTACAACAGTGAGCCAACTT
TRB_Jβ1.4r	TCRB	CATACCCAAGACAGAGAGCTGGGTTC
TRB_Jβ1.5r	TCRB	CTTACCTAGGATGGAGAGTCGAGTC
TRB_Jβ1.6r	TCRB	CATACCTGTCACAGTGAGCCTG
TRB_Jβ2.2r	TCRB	CTTACCCAGTACGGTCAGCCT
TRB_Jβ2.6r	TCRB	CTCGCCCAGCACGGTCAGCCT
TRB_Jβ2.7r	TCRB	CTTACCTGTAACCGTGAGCCTG
TRB_Jβ2.1r	TCRB	CCTTCTTACCTAGCACGGTGA
TRB_Jβ2.3r	TCRB	CCCGCTTACCGAGCACTGTCA
TRB_Jβ2.4r	TCRB	CCAGCTTACCCAGCACTGAGA
TRB_Jβ2.5r	TCRB	CGCGCACACCGAGCAC

Primer set 2 for immunoglobulins (IG)

Primer Name	Receptor Type	Sequence
IGH_JCONSr	IGH	CTTACCTGAGGAGACGGTGACC
IGH_VH1FR1f	IGH	GGCCTCAGTGAAGGTCTCCTGCAAG
IGH_VH2FR1f	IGH	GTCTGGTCCTACGCTGGTGAAACCC
IGH_VH3FR1f	IGH	CTGGGGGGTCCCTGAGACTCTCCTG
IGH_VH4FR1f	IGH	CTTCGGAGACCCTGTCCCTCACCTG
IGH_VH5FR1f	IGH	CGGGGAGTCTCTGAAGATCTCCTGT
IGH_VH6FR1f	IGH	TCGCAGACCCTCTCACTCACCTGTG
IGH_VH1FR2f	IGH	CTGGGTGCGACAGGCCCCTGGACAA
IGH_VH2FR2f	IGH	TGGATCCGTCAGCCCCCAGGGAAGG
IGH_VH3FR2f	IGH	GGTCCGCCAGGCTCCAGGGAA
IGH_VH4FR2f	IGH	TGGATCCGCCAGCCCCCAGGGAAGG
IGH_VH5FR2f	IGH	GGGTGCGCCAGATGCCCGGGAAAGG
IGH_VH6FR2f	IGH	TGGATCAGGCAGTCCCCATCGAGAG
IGH_VH7FR2f	IGH	TTGGGTGCGACAGGCCCCTGGACAA
IGH_VH1FR3f	IGH	TGGAGCTGAGCAGCCTGAGATCTGA
IGH_VH2FR3f	IGH	CAATGACCAACATGGACCCTGTGGA
IGH_VH3FR3f	IGH	TCTGCAAATGAACAGCCTGAGAGCC
IGH_VH4FR3f	IGH	GAGCTCTGTGACCGCCGCGGACACG
IGH_VH5FR3f	IGH	CAGCACCGCCTACCTGCAGTGGAGC
IGH_VH6FR3f	IGH	GTTCTCCCTGCAGCTGAACTCTGTG
IGH_VH7FR3f	IGH	CAGCACGGCATATCTGCAGATCAG
